# MicroRNAs 146a and 147b Biomarkers for Colorectal Tumor's Localization

**DOI:** 10.1155/2014/584852

**Published:** 2014-03-30

**Authors:** Inés Omrane, Nadia Kourda, Nejla Stambouli, Maud Privat, Imen Medimegh, Amira Arfaoui, Nancy Uhrhammer, Karim Bougatef, Olfa Baroudi, Hassen Bouzaienne, Raja Marrakchi, Yves-Jean Bignon, Amel Benammar-Elgaaied

**Affiliations:** ^1^Laboratory of Human Genetics Immunology and Pathology, Faculty of Sciences,Tunis El Manar University, 2092 Tunis, Tunisia; ^2^Laboratory of Anatomy and Pathology, Charles Nicolle Hospital of Tunis, Tunisia; ^3^Laboratory of Diagnosis and Molecular Genetics, Centre Jean Perrin, Clermont Ferrand, France

## Abstract

The recently identified class of microRNAs (miRs) provided a new insight into cancer research, since abnormalities of members of microRNAs family have been found in various types of cancer. However, the relationship between five miRNAs (miR146a, miR155, miR21, miR135a, and miR147b) and colorectal cancer remains unclear. In the present study, we examined expression of these miRNAs in 25 pair-matched colon cancer tissues and normal colon mucosa. The expression levels of miR146a, miR155, miR21, miR135a, and miR147b were quantified by real-time PCR. We found that miR21, miR146a, and miR135a were all expressed at higher levels in colon tumors. On the other hand, miR146a and miR147b expressions are significantly higher in left colon compared to right colon. These two miRs, especially miR146a, seemed to be markers for the left colon tumors. Moreover, significant proportional and inverse correlations were found between miR expressions in tumor and healthy tissue, and the correlations profiles were different depending on cancer localization. Taken together, these results lead us to suggest the presence of different mechanisms regulating miRs expression and consequently their target genes in left and right colon. So the pathway of colorectal carcinogenesis would be different according to the site of the tumor.

## 1. Introduction

Colorectal cancer (CRC) is the third most common malignancy in the world, accounting for more than 1.2 million cases and 608,700 deaths per year [1]. Indeed the number of established risk factors for colorectal cancer continues to grow including genetic factors and environmental factors such as smoking, physical inactivity, and obesity. To fight against CRC and to proceed to its early detection, screening tests that differ in sensitivity, specificity, cost, and safety [2, 3] such as colonoscopy, sigmoidoscopy, fecal occult blood test (FOBT), and stool DNA test have been developed. Some of these tests could be a promising alternative in the future but there is a pressing need for new noninvasive biomarkers to improve early detection of CRC.

A large number of recent studies have shown the utility of microRNAs as cancer-related biomarkers, supported by the finding that some microRNAs display altered expression profiles in cancers compared to normal tissues [4].

MicroRNAs (miRNAs) are evolutionarily conserved, endogenous, small, and noncoding RNAs of 19–25 nucleotides in length that repress protein translation through binding to target messenger RNAs [5]. The biogenesis of miRNAs involves a complex protein system. RNA polymerase II transcribes miRNA genes, generating long primary transcripts (pri-miRNAs) [6]. Subsequently, the process to yield mature miRNAs involves two steps including RNase-III enzymes and companion double-stranded RNA-binding domain (dsRBD) proteins. In the nucleus, the RNase III-type enzyme Drosha processes the long primary transcripts (pri-miRNA), yielding hairpin precursors (pre-miRNA). The pre-miRNAs are then exported to the cytoplasm where the miRNA hairpin is cleaved by the RNase III protein Dicer within the RISC loading complex [7, 8]. The guide strand, which corresponds to the mature miRNA, is then incorporated into the RNA-induced silencing (RISC) complex. In normal cells, miRNAs and their transcriptional regulators usually form autoregulatory loops aimed at controlling their respective levels [7–9]. MicroRNAs are expressed in a tissue specific manner and represent crucial factors in the regulation of different pathways involved in cell differentiation, cell cycle progression, apoptosis, and metabolism [10].

Several miRNAs have been identified in humans, but much remains to be understood about their precise cellular function and role in the development of diseases [5]. MicroRNAs have been demonstrated recently to potentially play a significant role in human carcinogenesis by functioning either as oncogenes or as tumor suppressors through their interactions with target genes critical to the development and progression of various cancers, including colorectal cancer [11]. In particular, some miRNAs have been reviewed about their potential role in the pathogenesis of CRC and their relationships with development, treatment, and prognosis of CRC [10].

MicroRNA155 (miR155) gene located on chromosome 21q21 is involved in the process of carcinogenesis [12]. Indeed miR155 was found to be overexpressed in many neoplastic diseases, such as B-cell lymphoma, leukemia, and colon cancer [13]. In addition, miR155 has been shown to be a key factor for innate and acquired immune responses [14].

MicroRNA21 (miR21) gene located on chromosome 17q23.2 is involved in inflammation, at least in part, by modulating cytokines responses [15]. miR21 is also one of the first miRNAs to be described as an oncomiR, as most of the targets of miR21 are tumor suppressor genes. MicroRNA21 has been reported to be upregulated in several human malignancies, including pancreatic cancer, breast cancer, lung cancer, gastric cancer, and colorectal cancer [16, 17].

MicroRNA146a (miR146a) gene is located on chromosome 5q34. A recent study provided evidence that miR146a acts as a modulator of the innate immune response and of the adaptive immune response as well. Additional studies showed that miR146a expression is upregulated in thyroid cancer and breast cancer in contrast to pancreatic cancer and gastric cancer in which the expression of this miR is downregulated [18]. It is interesting to note that miR146a has been detected in CRC patients sera but not in healthy subjects sera [19]. However, there is no clear data on the expression of this miR in colorectal cancer tissue.

MicroRNA135a (miR135a) is encoded in the human genome by two copies, which are located in the first intron of the* stabilin 1* (STAB1) gene on 3p21 and in intron 5 of* rhabdomyosarcoma 2-associated transcript* (RMST) gene on 12q23. Some studies demonstrated elevated levels of miR135a in colorectal tumor cells [20] and others identified miR135a as regulator of the tumor suppressor gene* adenomatous polyposis coli* (APC) with a potent effect on Wnt pathway activity. These results suggest that upregulation of miR135a might be involved in CRC pathogenesis [21].

MicroRNA147b gene, which is located on chromosome 15q21.1, is involved in posttranscriptional regulation of gene expression by affecting both stability and translation of target mRNAs. Studies demonstrated the participation of mir147 in a negative feedback loop that is able to inhibit the proinflammatory response of macrophages to multiple TLR ligands [22]. Few studies have been performed on the expression of this miRNA but some of them have reported a downregulation of miR147 in colorectal cancer tissue [10, 20].

In the current study, we aimed to investigate and compare the differential expression of the five miRNAs (miR146a, miR155, miR21, miR135a, and miR147b) in colon cancer (CC) tissue and normal colon mucosa and their relationships to the clinicopathological factors in Tunisian patients and this is in order to assess their involvement in colon carcinogenesis.

## 2. Materials and Methods

### 2.1. Patients and Tissue Samples

Paired samples of colon cancer and adjacent normal mucosa in paraffin-embedded blocks came from 25 patients recruited from the Charles Nicolle Hospital of Tunis. They were classified on the bases of their histopathological profiles.

### 2.2. RNA Extraction

Total RNA was extracted from tumor and corresponding normal tissue in paraffin-embedded blocks using the miRNeasy FFPE tissue kit (Qiagen) following the manufacturer's instructions. Total RNA was eluted in RNase-free water and stored at −20°C. The concentration and the quality of RNA were detected by spectrophotometer and by bioanalyser Agilent.

### 2.3. Reverse Transcription and Real-Time PCR

Reverse transcription was performed using the miScript II reverse transcription Kit (Qiagen). Expression of miRNAs was measured using the miScript SYBR Green PCR Kit and miScript Primer Assays (Qiagen) according to the manufacturer's protocol. RNU6B-6 miScript Primer Assay was used as an endogenous control. For each sample, the standard control used is RNU6B-6 as endogenous control. RNU6B-6 corresponds to a small nucleolar RNA that was selected by a previous study from the best performing controls for miR quantification assays. Indeed, based upon its expression level, its relative abundance, and its stability, as determined by several tissues and cell lines analysis, RNU6B-6 is used as endogenous control snRNA chosen as candidate reference in several papers published in the literature on miR quantification [23, 24]. miRNA expression was quantified using the 2^−ΔΔCT^ method.

### 2.4. Statistical Analysis

For miR quantification performed by real-time PCR, we express our results relatively to endogenous RNA RNU6B-6; thus, it is possible to compare between samples. From this, we have compared the relative expression of each miR between groups that have been stratified according to epidemiological or clinical features. The different tests used for comparison are standard tests displayed by SPSS software and significance of the results is given by this software. The correlation between miR expression and colon cancer was assessed by paired samples *t*-test and by explorer test. All *P* values lower than 0.05 were considered statistically significant. Coefficient correlations were calculated using Spearman's rho test. Changes in miR expression between patient subgroups were determined using one-way ANOVA. Cases values with *P* < 0.05 were considered significantly different. Receiver operating characteristics (ROC) analysis was generated to tag expression of miR146a and 147b in colon tissue. The sensitivity and specificity of the optimum cut-off point were defined as those values that maximized the area under the ROC curve (AUC). All statistical tests were analyzed using SPSS software (version 20.0).

## 3. Results

The expression levels of five different miRNAs that were known to be deregulated in various cancers were quantified by real-time PCR analysis. We further established the CT values for these genes of interest, in colon tumor and normal mucosa.

In a first step, we analyzed the expression levels of miRNAs in tumor compared to normal mucosa (Table 1). The relative expression ratio *R* which is presented as the *n*-fold change in gene expression allowed comparing the overall level of each miRNA. The value of *R* > 1 represented miRNAs overexpression in colon cancer relative to paired normal mucosa. The result reveals that miR21, miR135a, and miR146a are upregulated in colon cancer tissue compared to normal tissue (Table 1). Indeed, miR21 is expressed about 7 times more in tumor tissue than in healthy tissue (*P* = 0.011) and miR135a shows 8-fold overexpression in tumor tissue as compared to control tissue (*P* = 0.002), while miR146a overexpression is less high (4.02-fold) (*P* = 0.032). The relative expression ratio for miR155 suggests that it is also overexpressed in tumor tissue (2.84-fold) but statistical analysis did not carry out a significant difference in its expression between compared tissues (*P* = 0.087). The level of miR147b in colon cancer tissue compared to the healthy tissue tends towards downregulation (0.87-fold) (Table 1). However, this change was not statistically significant (*P* = 0.793).

To investigate the clinical significance of miR21, miR146a, miR135a, miR147b, and miR155 expression, the relationships between this expression and clinicopathological parameters of colon cancer patients were assessed. As shown in Table 2, level of each miR did not appear significantly associated with sex, tumor architecture, differentiation, histology, lymph node, or TNM stage.

**Figure 1:**
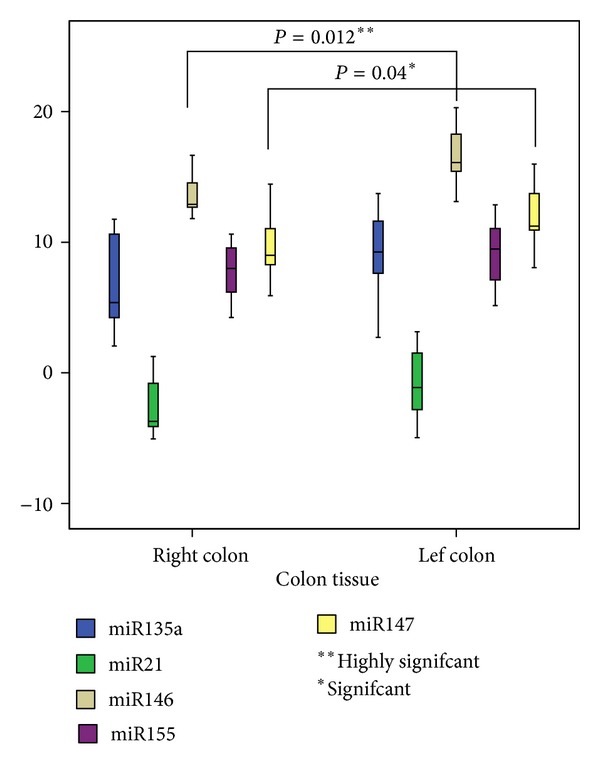
Comparison between miR expression levels according to tumor location.

According to tumor location, miR146a and miR147b showed a significant association with this feature in contrast to miR135a, miR21, and miR155 (Figure 1). We noticed specially that miR146a expression is significantly higher in left colon compared to right colon (*P* = 0.012) (Figure 1).

**Figure 2:**
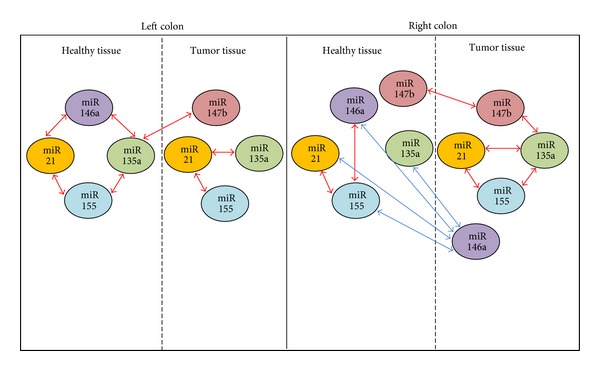
Correlation between expression miRs in tumoral tissue and healthy tissue according to localization.

Considering these results we have then evaluated the correlation between the expression of these miRs according to the tumor localization (Table 3 and Figure 2). As shown in Figure 2, correlation profile between miRs within a given tissue changes according to location (left or right colon) and status (healthy or tumoral tissue).

In these profiles the correlation between miR21 and miR155 is a constant feature. One has to notice that in healthy tissue correlation profiles in right and left colon are different indicating that regulation of gene expression is normally different according to left or right localization.

In both healthy tissues, miR147b does not correlate with the other miRs studied.

Interestingly, in tumor tissue we showed a correlation between miR147b and miR135a in right colon (*P* = 0.008) and the dissociation of miR146a expression from the other miRs in left colon (Table 3).

When we compared miRs expression correlations between normal and tumor tissues according to location, we found out that miR135a expression in normal left colon is correlated with miR147b expression in tumor (*P* = 0.031) (Table 3).

In right colon, dysregulation of miRs in tumor tissue as compared with normal tissue revealed that mir146a expression in tumor is inversely correlated with that of miR146a (*P* = 0.003), miR155 (*P* = 0.04), miR135a (*P* = 0.02), and miR21 (*P* = 0.02) in normal tissue, indicating a significative change of miR146a expression in right colon tumor (Table 3).

This analysis confirms the specific behavior of miR146a and miR147b in right and left colon.

In normal tissue, miR146a expression is linked to other miRs but is dissociated from their expression in tumors in both locations. On the contrary, miR147b is not linked to other miRs in normal tissues but its expression became associated with miR135a in right colon tumor (*P* = 0.008) and seemed to be in part determined in left tumor by that of miR135a in normal left colon (*P* = 0.03) (Table 3).

**Figure 3:**
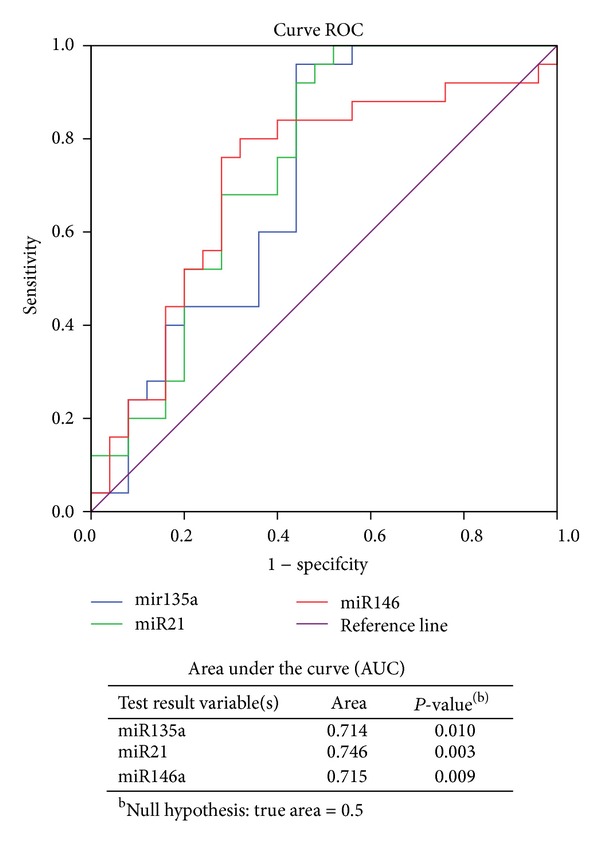
Evaluation of miR135a, miR146a, and miR21 expression in colorectal cancer tissue by curve ROC.

According to this work, we considered miR135a, miR146a, miR21, and miR147b as potential markers in colon cancer and performed ROC curves in order to evaluate the pertinence of their use. We have scored the expression of miR135a, miR146a, and miR21 by curve ROC to confirm their association with colorectal cancer by measuring their degree of sensibility and specificity with respect to this type of cancer (Figure 3). MiR135a could be a marker of colorectal cancer from a cut-off value of 9.35 with a sensitivity of 96% and a specificity of 56% and an AUC value of 0.71. In addition, miR21 and miR146a are significantly overexpressed in tumor tissue comparatively to healthy tissue from a cut-off value of −1.04 and 16.74, respectively, with a sensitivity of 68% and 76%, respectively, and a specificity of 72% for both miRs (Figure 3).

**Figure 4:**
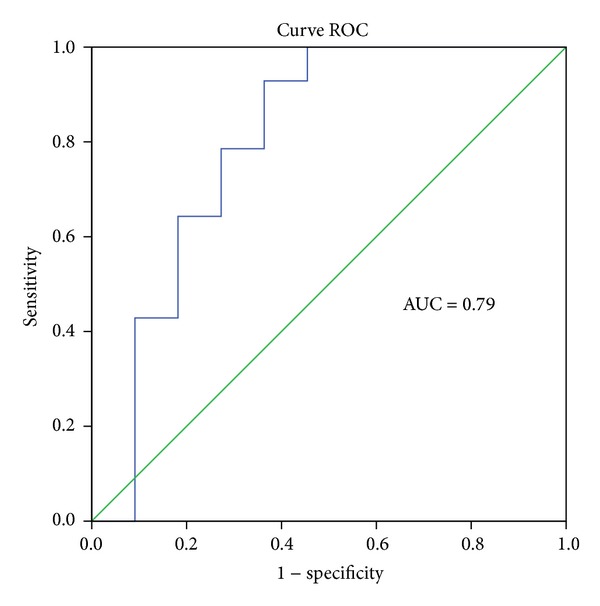
Evaluation of miR146a expression in left colon tissue by curve ROC.

Moreover, it appears that miR146a could be considered as a marker of the left colon from a cut-off value of 14.76 with a sensitivity of 78.6% and a specificity of 73% and an AUC value of 0.79 (Figure 4).

**Figure 5:**
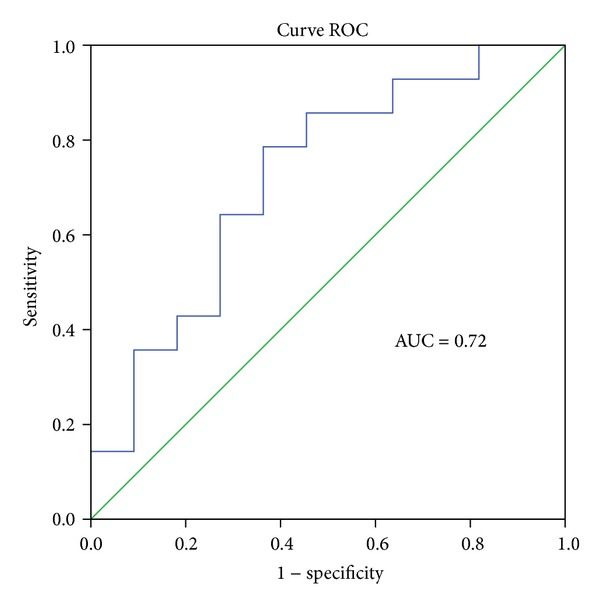
Evaluation of miR147b expression in left colon tissue by curve ROC.

Despite the expression of miR147b being not significantly different between healthy tissue and tumor tissue, it was found that expression of this microRNA is different according to tumor location. Indeed, miR147b as miR146a is significantly overexpressed in left colon comparatively to right colon (*P* = 0.04) (Table 2) from a cut-off value of 10.84 with a sensitivity of 78.6% and a specificity of 63.6% and an AUC value of 0.72 (Figure 5).

## 4. Discussion 

MicroRNAs are considered as potential molecular biomarkers for human malignancies. Indeed, they have an important role in differentiation, proliferation, and apoptosis. Both up- and downregulation of specific miRs have been described in CRC carcinogenesis [19]. Connection investigation between miRNAs and CRC aimed to better understand the colorectal carcinogenesis. In fact, it has been shown that miRs regulate expression of several oncogenic and tumor suppressor proteins such as the EGFR and Wnt/*β*-catenin pathways that are involved in pathogenesis and signaling pathway of CRC. Hence, miRs expression profile analysis enables us to predict prognosis, support diagnosis, and improve therapy responses in cancer [19, 25]. Indeed, miR profiles allowed us to classify human cancer of unknown primary origin as well as poorly differentiated tumors [25, 26]. Therefore, the microRNA profiles can potentially be used to develop predictive biomarkers for cancer.

In our study, we investigated expression profiles of five microRNAs (miR21, miR146a, miR135a, miR155, and miR147b) in tumor colon tissue compared to healthy adjacent tissue. We showed that miR21, miR146a, and miR135a are overexpressed in tumor tissue comparatively to control tissue (*P* = 0.011; *P* = 0.032; and *P* = 0.002, resp.). This result is in agreement with previous studies that have shown that miR21 is an oncogenic microRNA implicated as an inflammatory mediator and may promote inflammation-associated colon carcinogenesis [27]. Upregulation of miR21 and miR135a in CRC tissue and of miR146a in serum of patients with CRC [16, 17, 19, 20] has been also reported. There is no clear data in literature on the expression of miR146a in colorectal cancer tissue. Nevertheless, some studies have focused on genetic variability of this miR. In fact, presence of polymorphism in the gene encoding miR146a may contribute to colorectal cancer susceptibility in Chinese and in European Caucasians populations [28, 29]. Despite the relative expression suggesting that miR155 is overexpressed in tumor (2,84-fold), the statistical analysis showed no significant differential between the two types of specimens (*P* > 0.05). Contrary to our results, miR155 is overexpressed in colorectal cancer and in various tumors, including breast, lung, and pancreatic cancers [13, 30–32].

On the other hand, we showed that miR147b is not significantly downregulated in tumor colon tissue compared to healthy tissue. On the contrary, some studies have reported significant downregulation of miR147b [10, 20]. Other studies found that miR147 is regulated through toll-like receptor (TLR) signalization pathway depending on both nuclear factor *κ*B (NF*κ*B) andinterferon regulatory factor 3 (IRF3). Induction of miR147 expression by TLR is capable of downregulating excessive inflammatory responses [22].

Taken together these data indicate that miR135a, miR146a, and miR21 could be considered as a marker of CRC tumor with good specificity and sensitivity.

Further, we explored the relationship between expression of these five miRs and colon cancer stratified by tumor aspect, tumor differentiation, tumor histology, TNM stage, lymph node, and metastasis. We found no significant association or any significant differential expression of each miR according to clinical data. In fact, contrarily to our finding, other studies considered miR21 as biomarker for advanced stage of CRC, since its expression level was significantly higher in the late stages of the disease [33]. In addition, miR21 is implicated in cellular outgrowth, migration, invasion, and metastasis; it has also been considered as oncomiR targeting transcripts encoding regulator cell proliferation and apoptosis such as proteomics and programmed cell death protein 4 (PDCD4) and Sprouty2 (SPRY2 affects cellular outgrowth, branching, and migration) as well as phosphatase and tensin homologue (PTEN) [33, 34]. For miR135a, no association with disease presentation has been carried out in this study while it has been suggested by a previous study that miR135a may promote the growth and invasion of CRC cells through targeting APC and metastasis suppressor 1 (MTSS1) [21, 35]. In fact, Nagel et al. demonstrated that miR135a/b suppresses APC expression even if APC mutations are present [21]. Moreover, high expression levels of miR155 have been found to correlate with poor prognoses of lung cancer and pancreatic tumor [32, 36].

The differences observed between this study and previous works could be related to environmental conditions as well as to factors involved in miRs expression and their dysregulation in carcinogenesis and CRC progression. These factors, such as way of life and intestine microorganisms, dramatically vary among populations.

According to tumor location, we found that miR146a and miR147b are both more expressed in left colon tumors than in right colon tumors (*P* = 0.012 and *P* = 0.04, resp.). This suggests that miR146a and at a lesser extent miR147b may be biomarkers for a subtype of colon cancer tissue. The other miRs are expressed in the same way in the right or left colon (*P* > 0.05). In fact, we showed a great difference between profiles of miRs expression correlation. The difference between expression levels of each miR and the correlation coefficient between miR expressions could be due to the mechanisms of regulation related either to genetic factors or to epigenetic factors or both. One has to notice that in normal right and left colon, miRs expression profile is different leading to the suggestion that cells at the two locations do not have the same gene expression profile. Moreover, in the past two decades, a growing amount of data has been reported suggesting that carcinomas of the right and left colon should be considered as different tumor entities. In fact, right- and left-sided lesions exhibit different genetic, biological, and demographical characteristics and risk factors, suggesting that the carcinogenetic mechanism and progression of colon cancer lesions may differ with tumor location [37, 38]. The frequency of microsatellite instability (MSI) was significantly higher in right-sided colorectal cancers than in left-sided colorectal cancers [39–41]. On the other hand, the higher incidence of p53 mutations is predominantly present in left side [41–44]. A high level of epidermal and transforming growth factors EGF/TGF was also seen in left-sided tumors than right-sided [37]. In the present work we present data indicating that some miRNAs are markers in CRC according to location. Indeed, miR146a and miR147b could be considered as marker of left colon tumors with good specificity and sensitivity. In fact, cancers of the right and left colon differ in their epidemiology, molecular genetics, and behavior [45]. At the epidemiological level, left colon cancers are more frequent than right colon cancers, in high CCR geographic region. In the contrary countries with weak incidence, right colon cancers are more frequent [46, 47]. These epidemiological differences indicate that different risk factors combinations are responsible for each form of cancer. Secondly, the left colon is reached late during the digestion process which probably leads to difference as compared to right colon, in waste composition and, specially, in microbial composition. At cellular level, it appeared from our results that normal intestinal cells do not display the same miR profile in right and left colon, indicating that these cells do not have the same molecular expression profile. In fact, it has been shown that cell loss by apoptosis associated with the expression of a proapoptotic regulatory protein, Bak, is higher in the left than the right colon [48]. Hence, at physiological level and from the morphogenetic point of view intestinal cells are not identical in right and left colon. At genetic level, allelic losses and deletions 18q are more frequent in the left colon tumor than in the right colon. In contrast, right-sided cancers are relatively more common in hereditary nonpolyposis colon cancer than in sporadic cancer [49, 50]. All these features lead us to suggest that left colon cancerogenesis could be different from right colon; they would constitute different clinical entities.

On the other hand, from miRs dysregulation, it can be predicted which targets genes are involved in the carcinogenesis pathway. Few studies have focused on correlation between microRNA and targets. Zhong et al. confirmed gene Smad4 as predictive target of miR146a and showed downregulation of miR146a associated with upregulation of Smad4 at protein level. These data suggested that miR146a might influence proliferation through TGF-*β*1/Smad signal transduction pathway [51]. Shibuya et al. showed significant inverse correlations between miR21 and PDCD4 mRNA target, as well as between miR155 and tumor protein P53 inducible nuclear protein 1 (TP53INP1) mRNA target in colorectal cancer [52].

## 5. Conclusion

Our study shows upregulation of miR21, miR135a, and miR146a in colon cancer tissue compared to healthy colon tissue. We showed also differences between correlations of miR expression in right- and left-sided colon cancers suggesting that they are differently regulated and that there are different mechanisms of carcinogenesis between the two sites of the colon. Particularly interesting is the miR146a; its expression and role are unclear in several cancers and especially in colon cancer. In addition, overexpression of this miR appears to be specific to the side of colon tissue.

Further studies will be necessary to understand the biological role of these miRNAs in colon cancer in order to use them as potential targets for cancer prognosis and therapy.

## Figures and Tables

**Table 1:** Comparison of miRs expression levels in normal and tumoral colon tissues.

MicroRNAs studied	Patients number	Tumor tissue CT value (mean ± SD)	Normal tissue CT value (mean ± SD)	Expression ratio *R*	*P* value
miR21	25	−1.111 ± 3.24	1.644 ± 3.06	6.72	**0.011**
miR146a	25	15.499 ± 2.82	17.545 ± 3.04	4.02	**0.032**
miR135a	25	8.983 ± 4.21	11.983 ± 2.49	8	**0.002**
miR155	25	9.036 ± 3.05	10.549 ± 2.76	2.84	0.087
miR147b	25	10.970 ± 2.44	10.800 ± 2.21	0.88	0.793

**Table 2:** Relationship between clinicopathological features and miRs expression in colon cancer tissue.

	Parameters					
	*N* = 25	*P* value				
MicroRNAs		miR146a	miR147b	miR135a	miR21	miR155
Gender						
Male	17 (68%)	0.67	0.27	0.60	0.70	0.31
Female	8 (32%)					
Tumor location						
Right colon	11 (44%)	0.012	0.04	0.73	0.81	0.74
Left colon	14 (56%)					
Tumor architecture						
UIB*	13 (52%)	0.85	0.28	0.43	0.33	0.87
UI/U/B*	12 (48%)					
Tumor differentiation						
Low/moderate	6 (24%)	0.45	0.33	0.11	0.84	0.48
High	19 (76%)					
Tumor histology						
Nonmucinous carcinoma	21 (84%)	0.25	0.42	0.10	0.18	0.23
Mucinous carcinoma	4 (16%)					
Lymph node						
Absent	11 (44%)	0.95	0.85	0.88	0.66	0.51
Present	14 (56%)					
TNM stage						
I-II	10 (40%)	0.77	0.45	0.95	0.58	0.90
III-IV	15 (60%)					

*UIB: ulcerative infiltrative tumor budding; UI: ulcerative infiltrative tumor; U: ulcerative tumor; B: budding tumor.

**Table 3:** Correlation between expression miRs in tumoral and healthy tissue according to localization.

	Right colon			Left colon		
	MicroRNAs	*R*	*P* value	MicroRNAs	*R*	*P* value
miR146a healthy tissue	miR146a tumoral tissue	−0.76**	0.003	miR21 healthy tissue	0.55*	0.022
	miR155 healthy tissue	0.70**	0.007	miR135a healthy tissue	0.60*	0.01

miR146a tumoral tissue	miR21 healthy tissue	−0.62*	0.02	—	—	—
	miR155 healthy tissue	−0.52*	0.04	—	—	—
	miR135a healthy tissue	−0.60*	0.02	—	—	—

miR147b tumoral tissue	miR147b healthy tissue	0.58*	0.03	—	—	—
	miR135a tumoral tissue	0.70**	0.008	miR135a healthy tissue	0.50*	0.031

miR21 tumoral tissue	miR155 tumoral tissue	0.82**	0.001	miR155 tumoral tissue	0.80**	0.0003
	miR135a tumoral tissue	0.70**	0.009	miR135a tumoral tissue	0.70**	0.003

miR21 healthy tissue	miR155 healthy tissue	0.65*	0.01	miR155 healthy tissue	0.82**	0.0001

miR155 tumoral tissue	miR135a tumoral tissue	0.72**	0.006	—	—	—

miR155 healthy tissue	—	—	—	miR135a healthy tissue	0.80**	0.0002

*R*: Spearman's rho correlation coefficient; *correlation is significant at the 0.05 level; **correlation is significant at 0.01 level.
